# Fabrication Process Development for Optical Channel Waveguides in Sputtered Aluminum Nitride

**DOI:** 10.3390/mi16111259

**Published:** 2025-11-06

**Authors:** Soheila Mardani, Bjorn Jongebloed, Ward A. P. M. Hendriks, Meindert Dijkstra, Sonia M. Garcia-Blanco

**Affiliations:** 1 Integrated Optical Systems (IOS) Group, MESA+ Institute for Nanotechnology, University of Twente, 7500AE Enschede, The Netherlands; m.r.s.mardani@utwente.nl (S.M.); b.jongebloed@utwente.nl (B.J.); m.dijkstra@utwente.nl (M.D.); 2 ALUVIA Photonics, 7500AE Enschede, The Netherlands

**Keywords:** aluminum nitride (AlN) thin films, integrated photonics, optical waveguides

## Abstract

Aluminum nitride (AlN) is a wide-bandgap semiconductor (6.2 eV) with a broad transparency window spanning from the ultraviolet (UV) to the mid-infrared (MIR) wavelength region, making it a promising material for integrated photonics. In this work, AlN thin films using reactive RF sputtering are deposited, followed by annealing at 600 °C in a nitrogen atmosphere to reduce slab waveguide propagation losses. After annealing, the measured loss is 0.84 dB/cm at 978 nm, determined using the prism coupling method. A complete microfabrication process flow is then developed for the realization of optical channel waveguides. A key challenge in the processing of AlN is its susceptibility to oxidation when exposed to water or oxygen plasma, which significantly impacts device performance. The process is validated through the fabrication of microring resonators (MRRs), used to characterize the propagation losses of the AlN channel waveguides. The fabricated MRRs exhibit a quality factor of 12,000, corresponding to a propagation loss of 4.4 dB/cm at 1510–1515 nm. The dominant loss mechanisms are identified, and strategies for further process optimization are proposed.

## 1. Introduction

Aluminum nitride (AlN) is a highly promising material for photonic integrated circuits (PICs), owing to its unique combination of properties. It is CMOS-compatible and has a wide bandgap of 6.2 eV, allowing operation across a broad optical spectrum, from the ultraviolet (~200 nm) to the infrared [1]. In addition, AlN demonstrates useful Pockels, piezoelectric, and nonlinear optical coefficients, which, combined with sufficiently low optical losses, make it a versatile platform for a wide range of photonic applications [2,3]. These characteristics support the realization of both linear and nonlinear integrated photonic devices spanning a broad wavelength range.

AlN thin films can be deposited on various substrates using different deposition techniques, such as molecular beam epitaxy (MBE) [4,5], metal–organic chemical vapor deposition (MOCVD) [6,7], pulsed laser deposition (PLD) [8], and sputtering [9,10,11]. Early achievements in integrated AlN photonics were driven by polycrystalline thin films deposited using reactive magnetron sputtering, with groundbreaking contributions by Xiong et al. [12]. Sputter deposition produces highly uniform wafer-scale layers that are cost-effective and exhibit a high deposition rate facilitating large-scale photonics device fabrication. Minimizing waveguide propagation loss is an essential requirement for optical applications. In that study, the authors report propagation loss for different geometries of AlN waveguides between 0.6 and 1.9 dB/cm at 1550 nm wavelength [2,12]. Recently, losses as low as 0.15 dB/cm in the C-band for uncladded polycrystalline waveguides deposited by DC sputter deposition have been reported [13].

The fabrication of low-loss AlN waveguides requires not only high-quality film deposition [14], but also a process flow that preserves the properties of the AlN. Aluminum nitride is prone to oxidation when exposed to oxidative media. While reactive RF sputtering provides dense, polycrystalline AlN films with low slab propagation loss, subsequent fabrication steps such as chemical mechanical polishing (CMP), resist removal, and cladding deposition can introduce unwanted oxidation due to exposure to water or oxygen-rich environments. To prevent unwanted oxidation, the process must be carefully engineered to eliminate or modify such steps, thereby preserving the optical properties of the material while enabling reproducible, low-loss photonic devices with the desired functionality.

In this work, we investigate how the composition of aluminum nitride (AlN) thin films is affected by oxidative steps commonly encountered in standard microfabrication process flows. Based on this study, we propose a fabrication process that preserves the composition and structure of the AlN films, critical for retaining the desirable properties of the material for integrated photonics. To validate the process, we fabricate microring resonators and use them to characterize the propagation losses of cladded channel waveguides. A propagation loss of 4.4 dB/cm is measured for the fundamental TE mode at 1550 nm. We further analyze the origin of these elevated losses and suggest strategies for performance improvement.

## 2. Fabrication Process Flow

### 2.1. Aluminum Nitride Film Sputter Deposition and Annealing

Aluminum nitride (AlN) thin films were deposited on 100 mm silicon wafers coated with an 8 µm thick thermal oxide layer, SiO_2_, using two sputter deposition systems. In the first one, a radio frequency (RF) reactive sputtering system, TCOater (ILP), was utilized. The substrate temperature was maintained at 400 °C during deposition. A 3-inch high-purity (99.999%) aluminum target was powered by an RF supply operating at a constant power of 500 W. The chamber was evacuated to a base pressure of 7 × 10^−7^ mbar, and deposition was carried out at a stabilized working pressure of 5 × 10^−3^ mbar. Argon and nitrogen were used as the sputtering and reactive gases, with flow rates of 40 sccm and 4 sccm, respectively. These conditions yielded a deposition rate of 7.7 nm/min, resulting in an AlN film thickness of 153 ± 12 nm. The samples prepared in this sputtering system were utilized for the fabrication of the channel waveguides in this work.

An AJA ATC 1500 system was utilized to prepare the sample used to verify the effect of oxygen plasma on the depth composition of AlN thin films. Both 10 cm diameter silicon wafers with an 8 µm thick thermal oxide layer were used as the substrate and were mounted on a rotating holder. The deposition chamber was pre-evacuated with a turbomolecular pump, reaching a base pressure of 4 × 10^−7^ mbar. Two-inch aluminum targets powered by RF sources were used for sputtering, with RF power set to 200 W. Argon and nitrogen gas flows were maintained at 30 sccm and 7.9 sccm, respectively. The substrate holder was heated to 700 °C, and the process pressure was stabilized at 6.7 × 10^−3^ mbar to ensure high-quality film growth. This process produced a 195 nm thick AlN layer.

To enhance the optical quality of the AlN layer, a post-deposition annealing step was conducted at 600 °C in a nitrogen atmosphere. This thermal treatment was chosen based on previous findings [14], which demonstrated its effectiveness in reducing optical slab propagation losses by more than half at 633 nm.

**Figure 1:**
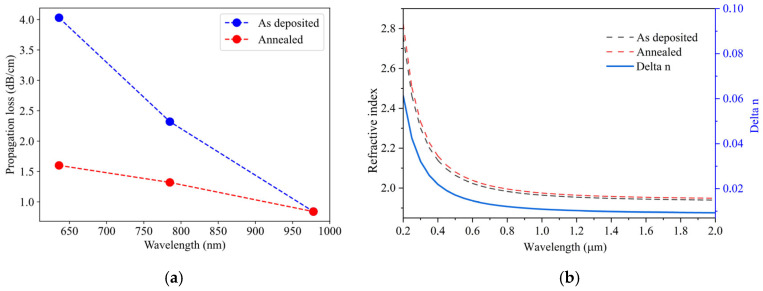
(**a**) Slab propagation losses before and after annealing of the film; (**b**) refractive index as a function of wavelength extracted from ellipsometry measurements for both the as-deposited as well as the annealed film (right axis) and the change in refractive index induced by the annealing (left axis).

The slab propagation losses were measured before and after annealing with the prism coupling method [15,16,17] using a Metricon 2010/M. Figure 1a shows that the propagation losses are reduced after annealing at 600 °C. The decrease in propagation losses is more prominent for shorter wavelengths, with a decrease in slab losses from 4.03 dB/cm down to 1.60 dB/cm at 633 nm and from 2.32 dB/cm to 1.32 dB/cm at 785 nm. The measured slab propagation losses at 978 nm of 0.86 dB/cm would lead to ~0.21 dB/cm slab loss at 1550 nm.

The decrease in propagation losses effect can be ascribed to changes in surface roughness. The decrease in surface roughness, induced by the annealing process, changes the scattering losses, which, in general, are more prominent at smaller wavelengths [18,19,20]. To support this claim, the root mean square (RMS) was measured with an atomic force microscope. The roughness of the surface of the as-deposited film was 0.73 ± 0.18 nm. After annealing, the surface roughness decreased to 0.68 ± 0.09 nm.

Ellipsometry measurements were performed to obtain the refractive index information of the deposited films in the wavelength range from 200 nm to 2000 nm. Figure 1b shows the measured refractive indices before and after annealing at 600 °C in a nitrogen atmosphere for three hours. From these ellipsometry measurements, the film thickness is found as well. The thickness and refractive index at 600 nm wavelength of the as-deposited AlN film are 153 ± 12 nm and 2.02 ± 0.01, respectively. After annealing, the layer thickness and refractive index are 145 ± 19 nm and 2.04 ± 0.02, respectively. These results indicate that annealing at 600 °C does not significantly change either the film thickness or the refractive index.

### 2.2. Effect of Oxidative Media on the Depth Composition of AlN Thin Films

One of the standard cleaning steps after channel waveguide etching is removing residual resist through oxygen plasma etching. The chemical stability and surface composition of AlN are highly sensitive to exposure to oxidative environments, making this a key concern in the fabrication of low-loss photonic and piezoelectric devices. As highlighted in the comprehensive review by Olivares et al. [21], exposure of AlN to oxygen-based plasmas results in the formation of a stable, involatile aluminum oxide (Al_2_O_3_) layer on the surface. To evaluate how suitable standard oxygen plasma cleaning is for AlN-based waveguide fabrication, we investigated the effect of plasma exposure on the chemical composition in depth of sputtered AlN films. To that aim, the film deposited in the AJA sputtering system was treated with oxygen plasma using a TePla 300 system to reproduce the standard oxygen plasma cleaning process utilized to remove photoresist after reactive ion etching. The plasma cleaning involved a 10 min preheating in nitrogen (500 sccm) at 1.0 mbar and 800 W, followed by a resist-stripping step in oxygen (500 sccm) under the same conditions. The specific process parameters are summarized in Table 1.

**Figure 2:**
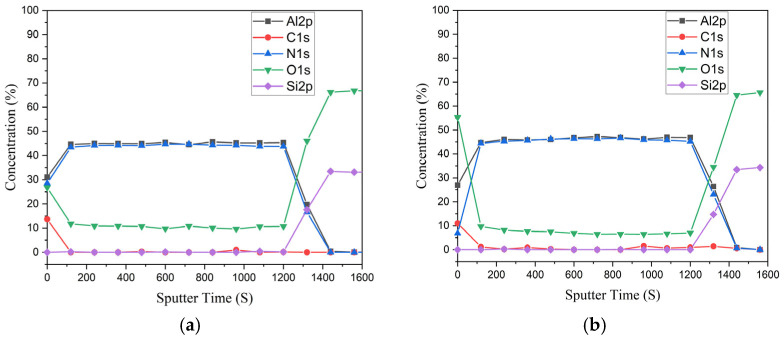
XPS depth profiles of as-deposited sputtered AlN thin films before (**a**) and after (**b**) O_2_ plasma exposure.

To determine the effect of this treatment on the depth composition of the sample, XPS depth profiling was conducted on both untreated and plasma-treated samples. It is important to note here that these layers were not subjected to the 600 °C annealing step prior to exposure to the oxygen plasma. Figure 2a,b show the atomic concentrations of Al, N, O, C, and Si as a function of argon etch time for the untreated and treated samples, respectively.

The XPS depth profiles provide a clear picture of the impact of O_2_ plasma treatment on the chemical composition of the sputtered AlN thin films of this work. As shown in Figure 2, the surface oxygen concentration significantly increased from approximately 27% in the untreated sample to 55% after plasma exposure. This confirms a substantial oxidation of the AlN surface during the cleaning process. Moreover, while the untreated sample showed ~10% oxygen content beyond the first ~20 nm, the treated sample exhibited a persistent oxygen signal of approximately 7–10% throughout the remaining depth of the film. This suggests that the oxygen plasma forms a superficial oxide layer, but the oxygen does not penetrate into the AlN bulk.

These findings indicate that although oxygen plasma treatment is effective for removing photoresist residues, it significantly oxidizes the surface. While this oxidation may not severely degrade performance in some photonic applications, it could negatively affect devices relying on pristine AlN properties, especially for nonlinear or electro-optic applications where purity and crystallinity are critical. Therefore, alternative cleaning methods to remove the photoresist after reactive ion etching that avoid bulk oxidation should be utilized to preserve the properties of the AlN core material.

High-temperature annealing was found to significantly change the depth composition of AlN thin films. As reported in our previous work, XPS depth profiling showed a substantial increase in oxygen incorporation throughout the AlN layer after annealing at 1150 °C in a nitrogen atmosphere [14]. This oxidation is due to the strong affinity of aluminum for oxygen and the presence of residual O_2_ and H_2_O in the annealing environment, which become highly reactive at elevated temperatures [22,23].

To minimize these effects and preserve the inherent Al–N bonds throughout the film, plasma cleaning and high temperature steps were avoided. Samples were cleaned using acetone and ethanol in an ultrasonic bath, without DI water. These organic solvents effectively remove residual surface contaminants without starting oxidation, maintaining the chemical integrity of the AlN surface for subsequent processing.

### 2.3. Final Process Flow for the Fabrication of Channel Waveguides in AlN

The annealed AlN film was stored in an N_2_ atmosphere before fabrication started to prevent oxidation of the layer. Fabrication starts by spin-coating a layer of negative resist (ARN7520.18). To prevent charging effects during electron beam lithography on the insulating substrate, a thin conductive polymer layer (AR-PC 5091.02) was subsequently spin-coated on top of the resist. Then, electron beam lithography was performed with the RAITH EBPG5150 to pattern the resist. After development of the resist, the waveguides were microstructured using reactive ion etching (RIE) in BCl_3_/HBr chemistry using an Oxford PlasmaPro 100 Cobra with 30 W RF power. BCl_3_ and HBr flows were set to 25 and 10 sccm, respectively. The selectivity of the AlN film etching with respect to the negative resist was approximately 1:1.8. After etching, the photoresist was stripped using Microstrip 5010 at 85 °C for 15 min. Afterwards, the sample was cleaned in an ultrasonic bath. First, the sample was placed in acetone for ten minutes, followed by ten minutes in ethanol, and then dried with nitrogen. To prevent oxidation, as explained above, we did not clean the wafer with oxygen plasma. To finalize the devices, a layer of 6 μm of SiO_2_ was deposited by PECVD as the top cladding on the AlN waveguides. The final step involved dicing the wafer into 10 × 10 mm^2^ chips.

### 2.4. Fabricated Waveguide Inspection

**Figure 3:**
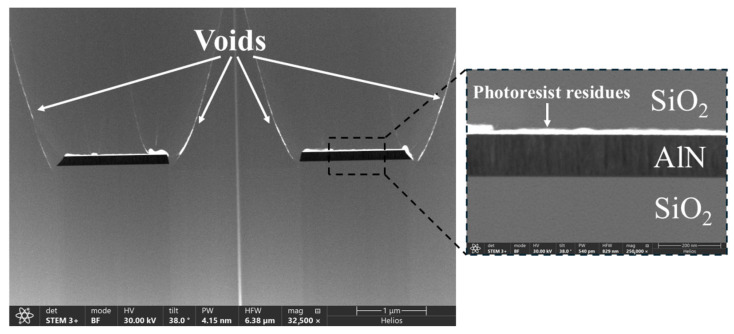
Bright field scanning transmission electron (STEM) image of the cross-section of the fabricated waveguide prepared by focused ion beam (FIB). The cross-section was taken across the directional coupler of the micro-ring resonator. Voids due to the non-conformality of the plasma-enhanced chemical vapor deposited (PECVD) SiO_2_ cladding are clearly seen, as well as the photoresist residues on the top surface of the waveguides, which are more clearly visible in the zoomed-in image on the right. The AlN core appears darker due to the higher density of aluminum nitride versus silicon dioxide.

The fabricated devices were inspected to verify whether the proposed process flow was leading to high-quality polycrystalline waveguides. A focused ion beam (FIB) was utilized to create cross-sections of the waveguides and subsequently inspect them with a scanning electron microscope (SEM) in bright field mode (Figure 3). Voids due to the non-conformal behavior of PECVD SiO_2_ are clearly visible at the edges of the waveguides. Such defects overlap with the waveguide mode. They are therefore expected to contribute to the propagation loss of the waveguides as scattering points. Charging artifacts are also visible on the top of the waveguides, which are linked to resist residues after microstrip cleaning. Such residues can also contribute to increased propagation losses.

Transmission electron microscopy measurements (TEM) were also performed to observe the crystallinity of the fabricated waveguides after the microfabrication process flow, since the promise of AlN for nonlinear and electro-optical applications strongly depends on its crystallinity.

**Figure 4:**
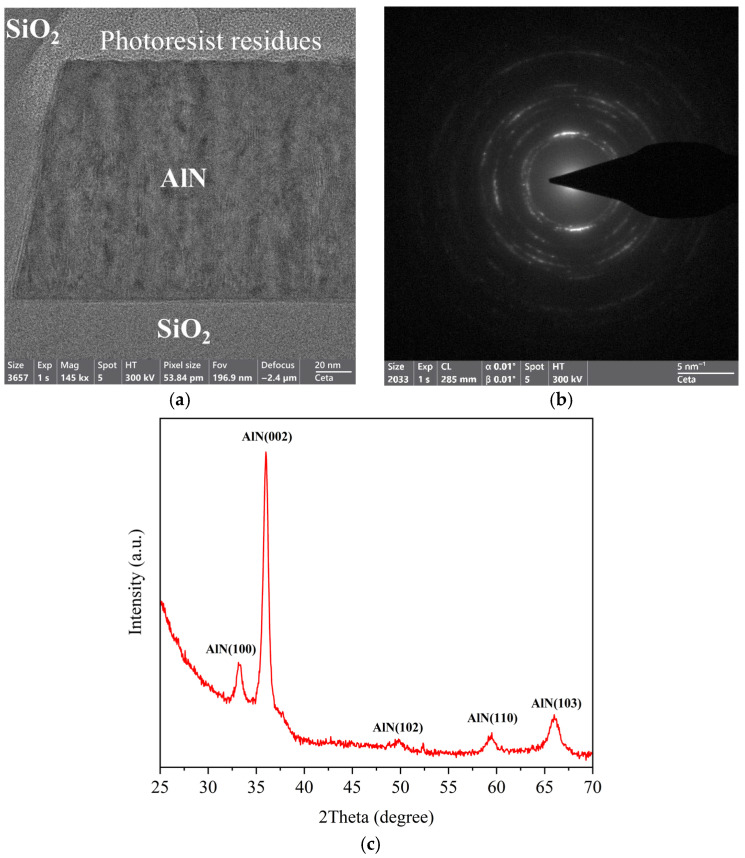
(**a**) Bright field transmission electron microscopy (TEM) image of an AlN waveguide cross-section, showing a preferred orientation within the polycrystalline matrix, (**b**) its diffraction diagram showing the polycrystalline nature of the material, and (**c**) XRD patterns of annealed AlN thin film.

A TEM image of a section of a waveguide, together with its diffraction diagram, is shown in Figure 4. The bright-field image in Figure 4a highlights the waveguide structure, from which key crystallographic properties can be extracted. The different shades of gray observed in the AlN core are due to the polycrystallinity of the material, which varies the scattering of electrons passing through the structure and being collected by the detector. The diffraction pattern shown in Figure 4b exhibits a concentric ring structure with discrete spots, indicative of a polycrystalline material [24]. The columnar grain structure observed in the bright-field image and the zoomed-in part of Figure 3 suggests a preferred orientation within the polycrystalline matrix. XRD measurements were performed on the annealed AlN film shown in Figure 4c. The diffraction pattern shows a dominant (002) peak at 36.1°, indicating a strong c-axis orientation. Additionally, the (100), (102), (110), and (103) peaks suggest a polycrystalline structure with contributions from multiple grain orientations, confirming the polycrystalline nature observed in the TEM measurements. From the TEM images, we can conclude that the process flow proposed in this work does not have a major influence on the structural properties of the AlN, and the AlN retains its polycrystalline nature throughout the waveguide fabrication process.

## 3. Optical Propagation Loss Characterization Using Micro-Ring Resonators

**Figure 5:**
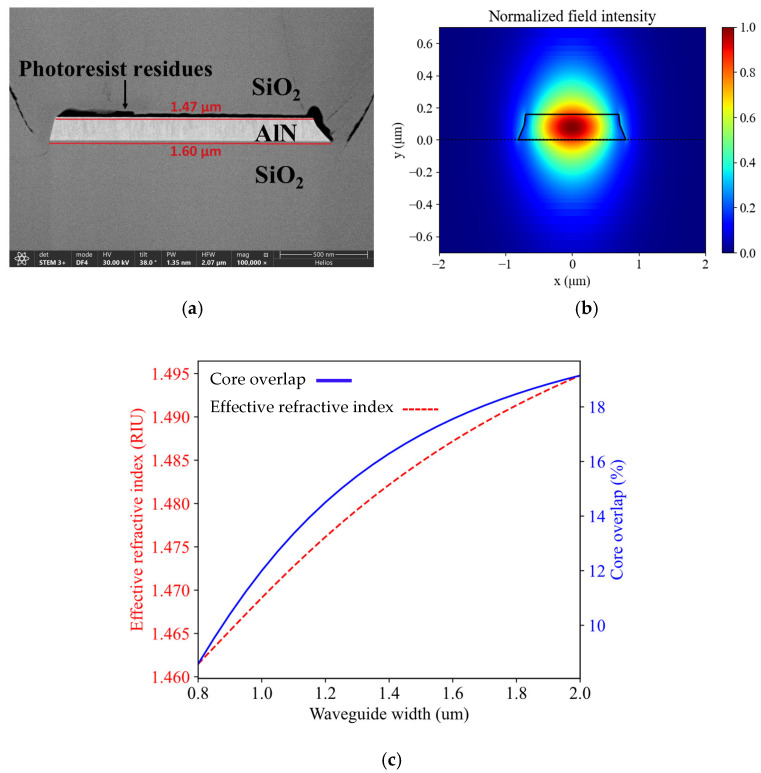
(**a**) Dark-field scanning transmission electron (STEM) image of the fabricated waveguide; (**b**) Geometry and optical mode of the simulated waveguide. Cross-section: 145 nm × 1.4 µm. Simulation wavelength: 1.55 µm; and (**c**) Simulated effective refractive index (red) and confinement factor of the fundamental TE mode (blue) as a function of waveguide width.

Simulations were performed using Lumerical MODE to calculate the effective refractive index and the confinement factor of the fabricated waveguides at the operating wavelength of 1550 nm. The waveguide geometry depicted in Figure 5a was analyzed. The layer stack consists of a 145 ± 19 nm thick AlN layer on top of an 8 µm buried oxide layer with 6 µm SiO_2_ top cladding. Figure 5b shows the optical mode profile of the waveguide fabricated in this work, with a width of 1.4 µm. The waveguide has a confinement factor of 17%. The confinement factor (i.e., overlap with the core of the waveguide) and the effective refractive index of the channel waveguides vary with the selected width, as can be seen in Figure 5c.

**Figure 6:**
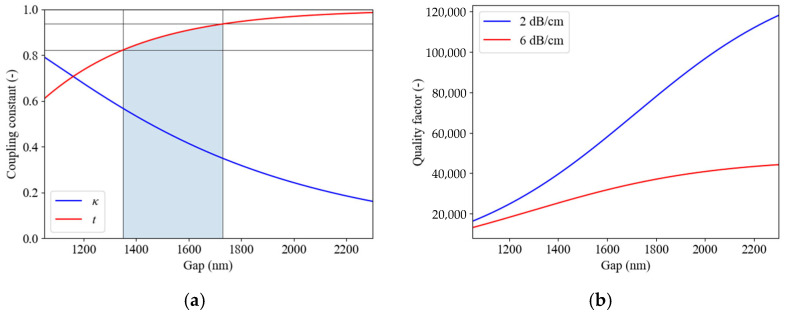
(**a**) Simulated coupling coefficient (blue) and transmission coefficient (red) as a function of gap that serves as a tool to select a proper range of coupling gaps, given an expected value of the propagation loss. Simulations performed for a wavelength of 1550 nm and waveguide dimensions of 145 nm × 1.4 mm; (**b**) Calculated Q-factor that would follow from the ring resonators in case of 2 dB/cm or 6 dB/cm propagation loss.

A microring resonator consists of a bus waveguide that is brought into close proximity of a microring that acts as a cavity [25]. These devices are commonly used in integrated photonics for applications such as filtering, modulation, or sensing [26]. MRRs are suitable devices for extracting propagation losses [27]. The spectral response of an MRR strongly depends on both the round-trip propagation losses in the cavity and the coupling between the cavity and the bus waveguide (i.e., the coupling losses from the cavity). The resonances in the transmission spectrum of the MRR can be fit with a Lorentzian curve, and from the fit, the full-width-at-half-maximum (FWHM), the quality factor (Q-factor), and free spectral range (FSR) are extracted. For a critically coupled ring resonator, i.e., a ring resonator for which the round-trip intrinsic electric field decay in the microring (*a*) equals the transmission coefficient of the coupler between the microring and bus waveguide (*t*), these properties can be used in a straightforward manner to calculate the losses [27]. For a ring cavity with a bend radius of 450 µm, both the coupling (*κ*) and transmission (*t*) coefficients of the coupler as a function of waveguide gap between the bus and ring waveguides have been calculated for 1550 nm wavelength and are shown in Figure 6a [28].

Based on the measured slab losses (i.e., 0.85 dB/cm at 978 nm of wavelength, which corresponds to ~0.21 dB/cm at 1550 nm), waveguide propagation losses are expected to be between 2.0 and 6.0 dB/cm, which corresponds to intrinsic electric field decay values, *a*, between 0.94 and 0.82. The expected waveguide propagation losses are larger than the film losses because of additional scattering losses caused by the roughness of the etched sidewalls and the presence of voids due to the non-conformal growth of the SiO_2_ claddings (Figure 2a). To achieve critical coupling, the coupling gap should be in the range from 1350 nm to 1700 nm, considering the expected propagation losses (i.e., the graded region in Figure 6a). Figure 6b includes the calculated quality factors expected for this loss range.

**Figure 7:**
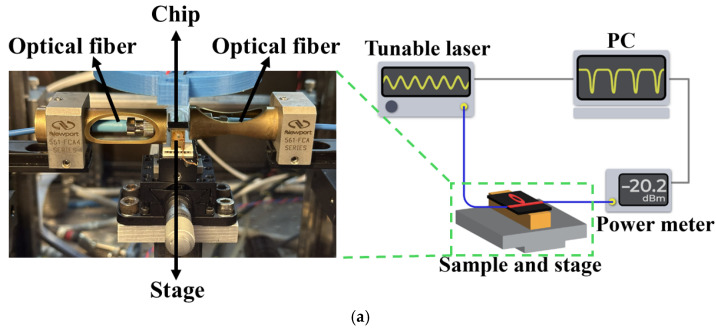

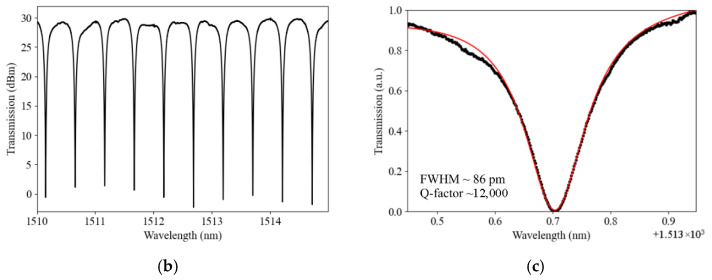
(**a**) Optical setup for measuring the transmission spectrum of the fabricated microring resonators, (**b**) measured transmission spectrum (black) of a microring resonator of radius 450 mm and gap of 1.6 mm for the range of wavelengths for which critical coupling is observed (i.e., from 1510 to 1515 nm) and (**c**) zoom in on one the resonances, including the Lorentzian fit (red) that is used to extract the propagation losses. The waveguide cross-section is 145 nm × 1.4 mm.

Figure 7a illustrates the optical characterization setup used to measure the transmission spectrum of the fabricated microring resonators. An Agilent 8164B tunable laser source was employed to sweep the wavelength range of interest, and the transmitted power was recorded using a power meter connected to a computer for data collection. Light from the tunable laser was coupled into the waveguide using a polarization-maintaining fiber patch cable (Thorlabs, P5-1550PM-FC-2). The same type of fiber was used at the output to collect the transmitted light. The input and output fibers were aligned to the chip facets with automated stages, which were adjusted to optimize coupling efficiency. An example of a full transmission spectrum for a microring with a designed gap of 1.6 µm, alongside the Lorentzian fit of one of these resonances, is depicted in Figure 7b,c. Critical coupling occurs for wavelengths around 1510–1515 nm. From this measurement, a Q-factor of 12,000 can be extracted, which corresponds to propagation losses of 4.4 dB/cm, which is in agreement with the calculations of Figure 3.

## 4. Conclusions

Although the measured losses are larger than the state-of-the-art losses in sputter-deposited AlN waveguides [1,2,12,29,30], several paths to reduce the losses of the current waveguides have been identified, including a longer cleaning step to ensure complete photoresist removal and increasing both the thickness and width of the AlN to decrease the overlap with the sidewalls while still remaining single mode. Substituting the PECVD SiO_2_ cladding with a more conformal ICP-PECVD or LPCVD SiO_2_ cladding would remove the voids observed at the edges of the waveguides [31], although further studies are required to determine the maximum temperature that the channel waveguides can sustain before degradation occurs [14]. These developments, together with the demonstration of the nonlinear as well as electro-optical properties of the resulting waveguides, are the subject of a follow-up ongoing study.

## Figures and Tables

**Table 1:** Oxygen plasma cleaning process parameters applied to the AlN thin film.

Step	O_2_ (sccm)	N_2_ (sccm)	Pressure (mbar)	Power (W)	Time (h:mm:ss)
Preheating	0	500	1.0	800	0:10:00
Stripping of resist	500	0	1.0	800	0:30:00

## Data Availability

The data supporting the reported results is available upon reasonable request.
